# The effects of serum leptin levels on thrombocyte aggregation in peritoneal dialysis patients

**DOI:** 10.12669/pjms.326.11021

**Published:** 2016

**Authors:** Serkan Bakirdogen, Necmi Eren, Sibel Gokcay Bek, Ozgur Mehtap, Mustafa Baki Cekmen

**Affiliations:** 1 Dr. Serkan Bakirdogen, Department of Nephrology, The Medical Faculty, Canakkale Onsekiz Mart University, Canakkale, Turkey; 2 Dr. Necmi Eren, Department of Nephrology, The Medical Faculty, Kocaeli University, Kocaeli, Turkey; 3 Dr. Sibel Gokcay Bek, Department of Nephrology, The Goverment Hospital, Kocaeli, Turkey; 4 Dr. Ozgur Mehtap, Department of Hematology, The Medical Faculty, Kocaeli University, Kocaeli, Turkey; 5 Dr. Mustafa Baki Cekmen, Department of Medical Chemistry, Medeniyet University, Istanbul, Turkey

**Keywords:** Peritoneal Dialysis, Thrombocyte Aggregation, Human Recombinant Leptin

## Abstract

**Objective:**

Serum leptin levels of chronic kidney disease patients have been detected higher than normal population. The aim of this study was to investigate the effects of serum leptin levels on thrombocyte aggregation in peritoneal dialysis patients.

**Methods:**

Fourty three peritoneal dialysis patients were included in the study. Thrombocyte aggregation was calculated from the whole blood subsequently the effects of different concentrations of human recombinant leptin on thrombocyte aggregations were investigated. Four test cells were used for this process. While leptin was not added into the first test cell, increasing amounts of leptin was added into the second, third and fourth test cells to attain the concentrations of 25, 50 and 100 ng/ml respectively.

**Results:**

Thrombocyte aggregation was inhibited by recombinant leptin in peritoneal dialysis patients. Thrombocyte aggregation mean values were found statistically significantly higher in first test cell when compared to leptin groups in peritoneal dialysis patients. For leptin groups we could not find any statistically significant differences for thrombocyte aggregation mean values between any of the groups.

**Conclusion:**

Further studies with larger number of peritoneal dialysis patients are required to prove the action of leptin on thrombocyte aggregation.

## INTRODUCTION

Cardiovascular diseases and their adverse events are the leading cause of morbidity and mortality in chronic kidney disease.^1^ In the development of atherothrombosis platelet aggregation and adhesion have critical importance.^2^ Leptin is a 16-kDa protein hormone synthesized from adipocytes. Its cell membrane receptor (Ob-R), is a member of the class 1 cytokine receptor family.^3^ Upon finding Ob-R in a megakaryoblastic cell line the relation between leptin and platelet functions was investigated.^4^ By Western blot analysis OB-Rb (the long form of its receptor) could be detected on platelet membrane also.^4^,^5^ The studies conducted so far in healthy (normal weight or obese) volunteers with in vitro leptin administration revealed varying results on aggregation of platelets activated by an agonist. While some studies have suggested that leptin increased platelet aggregation.^4^-^7^ one study reported no effect.^8^

Serum leptin level is increased in patients with end stage renal failure secondary to chronic inflammation, decreased glomerular filtration rate and increased insulin resistance.^9^ In a study by M.P. Fontan et al, higher serum leptin levels in peritoneal dialysis (PD) patients was compared to hemodialysis and predialysis stage 5 CKD patients.^10^ In our study we aimed to investigate the effect of varying concentrations of recombinant human leptin hormone on platelet aggregation in PD patients.

## METHODS

Fourty three PD patients followed between October 2011-April 2012 in the Kocaeli University Faculty of Medicine nephrology department of our institution either on continuous ambulatory peritoneal dialysis (CAPD) or automated peritoneal dialysis (APD) were randomly included in the study.

### Inclusion criteria

minimum PD duration of 3 months, consent of participation and age from 18 to 80.

### Exclusion criteria

patients with diabetes, platelet adhesion defects such as von Willebrand disease, Bernard-Soulier syndrome or platelet aggregation defects such as Glanzmann’s thrombasthenia, afibrinogenemia; history of an infectious disease in the last 10 days and use of at least one of following drugs in the last two weeks: acetylsalicylic acid, clopidogrel, ticlopidine, beta lactam antibiotics, corticosteroids and non-steroidal anti-inflammatory drugs. Approval from local ethics committee (Project No: 2011/64, Decision No: 7/2, Approval Date: 27.06.2011) and informed consent was taken from all participants before the study. Blood samples were taken following at least 8 hours of fasting. Serum leptin levels were determined by using ELISA method (Dynex-DSX / Virion-Serio (USA). Our work was planned to last for one year and in this time we only checked the serum leptin levels of the patients for once. ADP solution (Siemens PFA collagen / ADP Test Cartridge, Marburg / Germany) was added to the test cells to get the the final concentration of 10 µmole/L. Recombinant human leptin (OB) 5.0 mg (Biomyx Technology, San Diego / USA) was used.

### Multiple electrode platelet aggregometry

(MEA): It is a whole-blood impedance aggregometry method that allows the assessment of thrombocyte function. MEA was performed on the Multiplate analyser (Dynabyte medical, Munich, Germany), an instrument with five channels for paralel aggregometry measurements and a computer system for real-time analysis and documentation. Three criteria were calculated after the process in the device is completed. These are:

a) **Aggregation:** Reflects the increased electrical resistance during test process. It is expressed as aggregation unit (AU).
b) **Velocity:** Reflects the slope of the curve and is expressed as AU / minute
c) **Area under the curve (AUC**): Best marker of platelet aggregation, expressed as AU x minute

### Study design

Four test cells for each patient were prepared and process was carried aout for each cell in a sequential manner.

### 1. Test cell (C1)

Only 300 µL hirudinated blood, 300 µL normal saline and 31µL (10 µmole/L) ADP was put into C1 and platelet aggregation measurement was made.

For each of the other test cells (C2, C3, C4) in addition to content of C1, increasing amounts of leptin (25, 50 and 100 ng/ml respectively) solutions were added in a sequential manner and platelet aggregation measurement was made.

**Fig.1:**
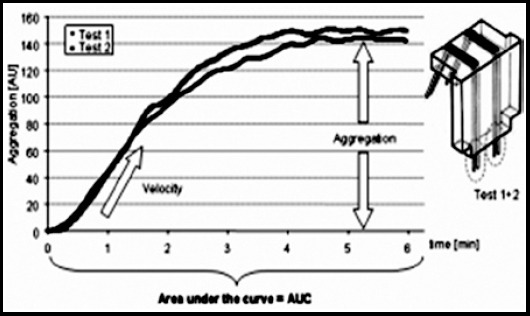
Aggregation-time graph.

In our study, due to lack of simultaneous serum leptin level measurements this parameter was not used in the evaluation. Aggregation-time graph used in the study is given in Fig.1^11^

### Statistical analysis

For statistical analysis of the study SSPS 15.0 software was used. Independent Samples t test was used for normally distributed variables. The other variables that do not comply with normal distribution were analyzed by Mann-Whitney U test. The relationship between four test cells was analyzed using paired t test and Wilcoxon test. Spearman correlation test was used to analyze the relation between platelet aggregation measurements of C1 and patients’ serum leptin levels and body mass indices (BMI). P values <0.05 were considered statistically significant.

## RESULTS

The study included 43 PD (95.3% CAPD, 4.7% APD) patients (21 male, 22 female). Etiology of CKD was given in Table-I. Biochemical findings and clinical characteristics of the patients are given in Table-II.

**Table-I:** Etiology of CKD in peritoneal dialysis patients.

Etiology	n (%)
Hypertensive nephropathy	23 (%53,5)
Chronic glomerulonephritis	11 (%25,6)
Autosomal dominant polycystic kidney disease	3 (%7)
Amyloidosis	1 (%2,3)
Urological causes	1 (%2,3)
Unknown	4 (%9,3)

Total	43 (100)

**Table-II:** Biochemical findings and clinical characteristics of the PD patients.

	Mean± Standard deviation
Age (year)	48±11,2
Dialysis duration (month)	40,7±22,8
BMI (kg/m^2^)	27±4
Serum leptin (ng/ml)	41,1±55,3
Serum BUN (mg/dl)	53,3±15
Serum albümin (g/dl)	3,5±0,4
Total Kt/V (week)	2,37±0,9
CRP (mg/dl)	0,81±0,86
PLT (mm^3^)	254332±72779
WBC (mm^3^)	7112±1779
Hemoglobin (g/dl)	10,5±1,9
Seum uric acid (mg/dl)	5,65±0,9
Serum parathormone (pg/ml)	568,4±637,6
Ferritin (ng/ml)	604,6±494,8

When mean serum leptin levels of PD patients were compared with platelet aggregation measurements in first test cell (C1), statistically significant difference was not detected for mean AUC, velocity and aggregation mean values (p values were 0.348, 0.186, 0.476 respectively). Similarly, when platelet aggregation measurements according to C1 results were compared with average BMI no statistically significant difference was found (p values were 0.999, 0.395, 0.576) between the patients. The mean platelet aggregation values of the patients and comparison of test cells are given in Table-III.

**Table-III:** The mean platelet aggregation values of PD patients and comparison of test cells.

Mean value	C1	C2	C3	C4
AUC (AUxminute)	825,00	513,03	508,10	557,71
Aggragation (AU)	152,713	91,194	90,034	97,882
Velocity (AU/minute)	17,832	12,416	12,483	13,386

P values of C1-C2, C1-C3 and C1-C4 for AUC and aggregation mean values were found 0.001, and p values of velocity mean value were found 0.016, 0.004 and 0.001 for the same compared test cells respectively. For leptin groups (C2, C3 and C4) we could not find any statistically significant differences for AUC, velocity and aggregation mean values between any of the groups.

When we divide PD patients, according to their serum leptin levels measured in the recent year as those above (n=16) and below (n=27) 25 ng/ml into two groups and compare their aggregation parameters with C1 we did not find increased platelet aggregation in higher serum leptin group (p=0,2 for AUC and aggregation, p=0,1 for velocity).

## DISCUSSION

In our study, platelet aggregation was measured using Multiplate device capable of measuring the whole blood. So far in the studies investigating the effect of leptin on platelet aggregation, the researchers have preferred using platelet rich plasma.^4^-^8^ But whole blood samples provide a more physiological environment for platelet aggregation measurement than other methods since other shaped elements along with platelets are present in whole blood.^12^ Monocytes and neutrophils form heterotypic aggregates with platelets via P-selectin molecules present on the surface of both leukocytes and platelets.^13^ Platelet-neutrophil interaction in whole blood especially in early phase may decrease platelets’ response.^14^ Although the mechanism is not fully understood, contact of platelets with erythrocytes increases the efficiency and functional capacity of them.^15^

Malyszko et al, in their study investigating the relationship between plasma and peritoneal fluid leptin levels with platelet aggregation by using ristocetin and arachidonic acid as agonists, found a positive correlation between these parameters.^16^ Differently, we searched in vitro effect of varying concentrations of human recombinant leptin on platelets activated with ADP in PD patients and we could not find a statistically significant difference for ADP activated platelet aggregation parameters in different leptin concentrations.

### Limitations of the study

Lack of simultaneous serum leptin determination, lack of data regarding soluble leptin receptor (sOb-R) and leptin resistance (leptin/sOb-R) and the lack of a control group of healthy volunteers.

In our study we detected that human recombinant leptin with a final concentration of 25 ng/ml reduced the aggregability of ADP activated platelets. We also found that this effect persisted in the higher concentrations of leptin (50 and 100 ng/ml) but not as a stronger inhibition in a dose-dependent manner. When we divide PD patients, according to their serum leptin levels measured in the recent year as those above (n=16) and below (n=27) 25 ng/ml into two groups and compare their aggregation parameters with C1 we did not find increased platelet aggregation in higher serum leptin group. These seemingly contradictory results might indicate that the effect of leptin on thrombocytes might differ in vitro and in vivo. While in the first case it persists in the circulation continuously, however in experimental models only the short term effect of it is measured, as in our study. J. Beltowski et al, reported natriuresis and nitric oxide (NO) like effects in their experimental study on rats for the short term effect of leptin when applied acutely. In the same study chronically induced hyperleptinemia was shown to induce resistance to natriuresis and NO like effects.^17^ Besides vascular tone regulatory effect, NO also reduces platelet adhesion and aggregation ability.^18^ In our study, inhibition of ADP activated platelet aggregation with in vitro administration of human recombinant leptin in PD patients can be explained by its NO-like immediate effect. Lack of a statistically significant relation between serum leptin levels and platelet aggeregation parameters in our PD patients might be explained by leptin receptor downregulation or disturbed postreceptor cytoplasmic pathways leading to resistance developped againist NO-like effects seen in chronic hyperleptinemia as sugeste by the literature.^17^

## CONCLUSION

In order to prove the effect of leptin on platelet aggregation in PD patients further studies with larger number of patients are needed.
